# Effect of aspirin on primary prevention of cardiovascular disease and mortality among patients with chronic kidney disease

**DOI:** 10.1038/s41598-022-22474-9

**Published:** 2022-10-22

**Authors:** Hadar Haim-Pinhas, Gil Yoskovitz, Michael Lishner, David Pereg, Yona Kitay-Cohen, Guy Topaz, Yaron Sela, Ori Wand, Ilan Rozenberg, Sydney Benchetrit, Keren Cohen-Hagai

**Affiliations:** 1 Department of Internal Medicine C, Meir Medical Center, Kfar Saba, Israel; 2 Sackler Faculty of Medicine, Tel Aviv University, Tel Aviv, Israel; 3 Department of Hematology, Meir Medical Center, Kfar Saba, Israel; 4 Department of Cardiology, Meir Medical Center, Kfar Saba, Israel; 5 The Research Center for Internet Psychology, School of Communications, Reichman University, Herzliya, Israel; 6 Department of Pulmonology, Barzilai University Medical Center, Ashkelon, Israel; 7 Faculty of Health Sciences, Ben Gurion University of the Negev, Beer Sheva, Israel; 8 Department of Nephrology and Hypertension, Meir Medical Center, 59 Tchernichovsky St., 4428164 Kfar Saba, Israel

**Keywords:** Cardiology, Cardiovascular diseases, Chronic kidney disease, Chronic kidney disease

## Abstract

Chronic kidney disease is associated with an increased risk for cardiovascular and bleeding events. Data regarding the effectiveness and risks of aspirin therapy for primary prevention in the high-risk group of patients with chronic kidney disease are scant and controversial. This retrospective study included patients with chronic kidney disease. Participants were divided according to aspirin use. Outcomes included non-fatal cardiovascular events, major bleeding events and all-cause mortality. Among 10,303 patients, 2169 met the inclusion criteria and 1818 were included after 1:1 propensity-score matching. Our final cohort included patients with mean age of 73.4 ± 11.6 years, estimated glomerular filtration rate of 31.5 ± 10.5 ml/min/1.73m^2^ with follow up of 4.9 ± 1.5 years. There were no significant differences in all-cause mortality and bleeding events (odds ratio = 1.03, confidence interval [0.62, 1.84], *p* = .58 and odds ratio = 1.09, confidence interval [0.65, 1.72], *p* = .87 respectively). The incidence of cardiovascular events was higher in aspirin users versus non-users on univariate analysis (*p* < 0.01) and was comparable after controlling for possible risk-factors (OR = 1.05, CI [0.61, 3.14], *p* = .85). Chronic aspirin use for primary prevention of cardiovascular disease was not associated with lower mortality, cardiovascular events or increased bleeding among patients with chronic kidney disease. Those results were unexpected and should prompt further research in this field.

## Introduction

Chronic kidney disease (CKD) affects 10%–16% of adults worldwide. Compared with the general population, individuals with CKD are at increased risk of cardiovascular diseases (CVD), cardiovascular death and all-cause mortality^1,2^. The adjusted hazard ratio (HR) for death and cardiovascular events is inversely related to the estimated GFR (eGFR)^1^.

Several potential mechanisms have been proposed for the elevated CVD risk in patients with CKD, including increased oxidative stress, endothelial dysfunction and extensive vascular calcifications, altered pharmacodynamic properties of antiplatelet agents and accelerated plaque progression and rupture^2,3^. Increased risk for drug-drug interactions, changes in renal and non-renal drug clearance, and underlying qualitative platelet defects with altered reactivity and aggregability from uremia may be other factors that increase risk for CVD and death in CKD patients^1,2,4^.

Paradoxically, CKD patients are not only at increased risk for CVD, but also for bleeding complications. In a 2016 administrative data study of > 500,000 adults with CKD, the risk of major hemorrhage gradually increased as the eGFR declined and albuminuria increased^5^. It is thought that the underlying cause is a complex hemostatic disorder, platelet-platelet and platelet-vessel wall abnormal interactions^2,3^. Thus, advanced kidney failure may cause two opposing hemostatic complications: bleeding diathesis and thrombotic predisposition.

Aspirin is one of the most frequently used medications for both primary and secondary prevention of CVD. It inhibits cycloxygenase-1 (COX-1) and decreases platelet thromboxane production. Among patients with cardiovascular disease, 28% exhibit low or no response to aspirin, termed aspirin resistance. Impaired renal function has also been associated with this phenomenon^1,3^. A 34.8% prevalence of aspirin resistance has been described in hemodialyzed patients^6^. Endothelial dysfunction in CKD patients with resultant abnormal prostacyclin and thromboxane production may reduce aspirin’s ability to inhibit COX-1 and may explain the higher prevalence of aspirin resistance in these patients^3^.

It is well-established that aspirin use is beneficial for patients with known CVD (secondary prevention), but its role in primary prevention among patients without known CVD, is less clear. The benefit of aspirin as primary prevention was evaluated in several populations, such as the elderly and those with diabetes^7–10^. The use of low-dose aspirin led to lower risk for serious vascular events than placebo among persons with diabetes but also to higher rates of major bleeding. Unexpectedly, the ASPREE investigator group found no decrease in cardiovascular events and higher all-cause mortality among apparently healthy, older adults who received daily aspirin than among the placebo group^7,11^.

Despite higher cardiovascular risk, the benefit of primary prevention in patients with CKD was not evaluated against placebo in randomized controlled trials, and results of observational studies are controversial. The current study assessed the efficacy and safety of chronic aspirin treatment among CKD patients without known CVD compared to patients who were not treated with aspirin.

## Methods

### Study design

This retrospective, observational study evaluated clinical outcomes of non-dialysis dependent CKD patients without known cardiovascular disease. Electronic medical records (EMR) of all patients with CKD treated at Meir Medical Center from January 2014 to December 2018 were evaluated. Meir Medical Center is a tertiary hospital affiliated to Tel Aviv University, is owned and operated by Clalit Health Services, which is the largest health service organization in Israel.

The EMR of all included patients are linked to the national death registry. Patients with available data who did not meet exclusion criteria were included in this analysis. Patients with no available data or EMR were excluded. Patients were followed until July 2020. All deaths were recorded using EMR of each patient individually (including deaths out of Meir Medical Center).

The study is reported according to the STROBE statement guidelines.

Data were collected from EMR using ICD coding.

### Participants

The study included adults ≥ 18 years of age with CKD who were hospitalized or treated in the ambulatory clinics at Meir Medical Center during the study period. CKD diagnosis was based on calculated eGFR < 60 ml/min/1.73m^2^. Patients treated with chronic anticoagulation (> 3 months), high-dose aspirin (> 300 mg/day) or anti-platelet treatment other than aspirin were excluded. Those with a documented diagnosis of CVD (ischemic heart disease, stroke, transient ischemic attack or peripheral vascular disease) and patients who received dialysis prior to study entry or who underwent kidney transplantation were also excluded.

### CKD stage

GFR was estimated individually for each patient using the CKD-EPI formula^12^. CKD-EPI is a widely-accepted, validated tool for estimating GFR. Participants were classified according to CKD stage as defined in the KDIGO 2012 guidelines^13^: stage 3A, mildly to moderately decreased eGFR (45–59 ml/min/1.73m^2^); stage 3B, moderately to severely decreased (eGFR 30–44 ml/min/1.73m^2^); stage 4, severely decreased eGFR (15–29 ml/min/1.73m^2^) and stage 5, kidney failure with eGFR < 15 ml/min/1.73m^2^ not treated by renal replacement therapy.

### Study groups

Patients treated with low-dose aspirin (75–100 mg per day) for at least 3 consecutive months were allocated to the aspirin group and those who were not treated with aspirin comprised the non-aspirin group. Aspirin use was confirmed for each patient based on follow-up of prescriptions.

### Outcome measures

Outcomes included non-fatal cardiovascular events, major bleeding events and all-cause mortality. Mortality data were collected for each patient from EMR.

Non-fatal cardiovascular events included acute coronary syndrome (ACS), stroke or transient ischemic attack, or peripheral vascular disease; Major bleeding events were defined as bleeding that required hospitalization and/or blood transfusion.

### Ethical issues

The study was approved by the Ethics Committee and Institutional Review Board of Meir Medical Center (no. MMC-0341-18). The committee waived the requirement for informed consent due to the retrospective nature of the study. The study was performed in accordance with the Declaration of Helsinki and Good Clinical Practice guidelines.

### Data analysis

The data were analyzed using SPSS software, version 25. To minimize methodological biases, and specifically confounding by indication, we conducted a Propensity Score Matching procedure. Therefore, aspirin uses and non-uses were matched on a 1:1 ratio according to age and sex. The propensity score, defined as the conditional probability of the baseline covariates, was calculated using a multivariate logistic regression model. A random matching order was used and replacements were not allowed.

Descriptive statistics are reported using means and standard deviations for continuous variables, and frequencies for discrete variables. Univariate comparisons were performed using Mann–Whitney tests for continuous variables, and Chi-square tests for discrete variables.

Multivariate models for predicting mortality, major bleeding, and CVD by aspirin use were conducted using logistic regression. Cox regression models were also conducted while controlling for possible risk-factors including age, sex and comorbidities such as hypertension, diabetes, atrial fibrillation, and eGFR.

Kaplan–Meier and adjusted cox analyses were conducted to compare mortality rates over time between the study groups. *p*-values < 0.05 were considered statistically significant.

## Results

**Figure 1:**
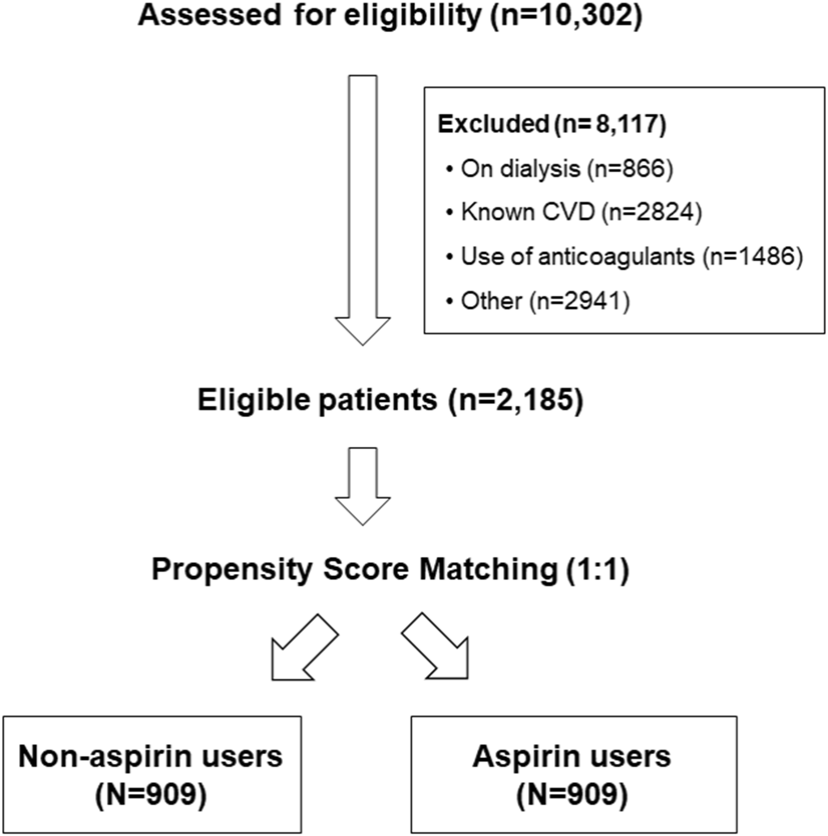
Flow chart of study participants. Electronic medical records of 10,302 patients with CKD treated at Meir Medical Center from January 2014 to December 2018 were evaluated. Excluded were 8,117 patients due to end-stage kidney disease treated by dialysis; previous *CVD* chronic use of anti-coagulants; chronic use of anti-platelet treatment that is not aspirin, or unavailable for follow-up were classified as other.

During the study period, 10,302 patients with CKD comprised the primary study cohort. Among them, 8,117 patients were excluded due to previous cardiovascular disease, anticoagulation or anti-platelet treatment, dialysis, or other causes as detailed in Fig. 1.

### Study population

A total of 2,185 CKD patients met the inclusion and exclusion criteria. Propensity score matching was done in order to match age and sex between aspirin users and non-user s on a 1:1 ratio, yielded 2 equal groups, with 909 patients in each. Before propensity score matching, patients in the aspirin group were significantly older than non-aspirin users. The baseline characteristics of the entire unmatched and propensity score-matched cohorts are presented on Supplementary (Table S1). After propensity score matching, age, sex and baseline kidney function were comparable between two groups (Table S1).

After propensity-score matching, mean age of the study cohort was 73.4 years and mean follow-up was 4.9 ± 1.5 years, both were similar between study groups. The average eGFR of the entire cohort was 31.5 ± 10.48 ml/min/1.73m^2^, and was similar between groups, as was the categorization according to CKD stages (Table 1).

**Table 1:** Clinical and demographic characteristics of study population.

Characteristic	Aspirin (N = 909)	Non-aspirin (N = 909)	Total (N = 1,818)	Chi-squared	Risk ratio [95% CI]	*p*-value
**Sex**				0.32	0.94 [0.78, 1.14]	.57
Male	454 (49.2)	468 (50.7)	922 (50.9)			
Female	439 (51.0)	439 (48.9)	896 (49.1)			
Age, years	74.29 ± 10.29	73.88 ± 12.78	73.43 ± 11.63			.47
Follow-up, years	4.83 ± 1.31	4.931 ± 1.48	4.85 ± 1.49			.43
First creatinine, mg/dL	2.24 ± 1.02	2.18 ± 1.04	2.21 ± 1.05			.75
Baseline eGFR, ml/min/1.73m^2^	32.56 ± 11.21	31.87 ± 11.26	31.52 ± 10.48			.72
**CKD group**				2.4		.76
Stage 3A	79 (8.7)	71 (7.8)	177 (9.8)			
Stage 3B	366 (40.5)	386 (42.5)	888 (49.1)			
Stage 4	355 (39.3)	342 (37.6)	642 (35.5)			
Stage 5	103 (11.4)	111 (12.2)	103 (5.7)			
Hypertension	790 (86.9)	778 (85.6)	1568 (86.2)	0.66	1.12 [0.86, 1.42]	.41
Diabetes mellitus	593 (65.2)	478 (52.6)	1071 (58.9)	30.4	1.69 [1.40, 2.04]	< .01
Atrial fibrillation	71 (7.8)	36 (4.0)	107 (5.9)	12.13	2.05 [1.36, 3.10]	< .01

Discrepancies between total number of patients, and number of patients for both groups are due to missing data.

Values are presented as absolute numbers (percentage) or as mean ± SD.

Rates of diabetes mellitus and atrial fibrillation were higher among aspirin users compared to non-users (65.0% vs. 52.6% for diabetes, Risk ratio (RR) = 1.69, *p* < 0.01 and 7.8% vs. 4.0% for atrial fibrillation, RR = 2.05, *p* < 0.01; Table 1).

### Outcomes

**Figure 2:**
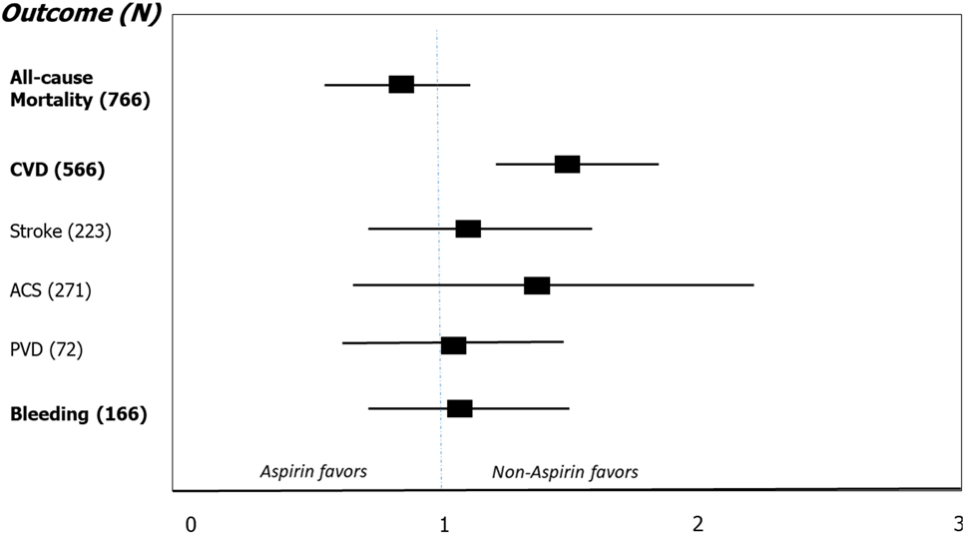
Forest plot of outcomes in study population (N = 1,818). Outcomes are presented as odds ratios with 95% confidence intervals. OR < 1 favors aspirin use, OR > 1 favors non using aspirin. Univariate analysis demonstrated higher incidence of cardiovascular events among aspirin users (*p* < 0.01).

**Figure 3:**
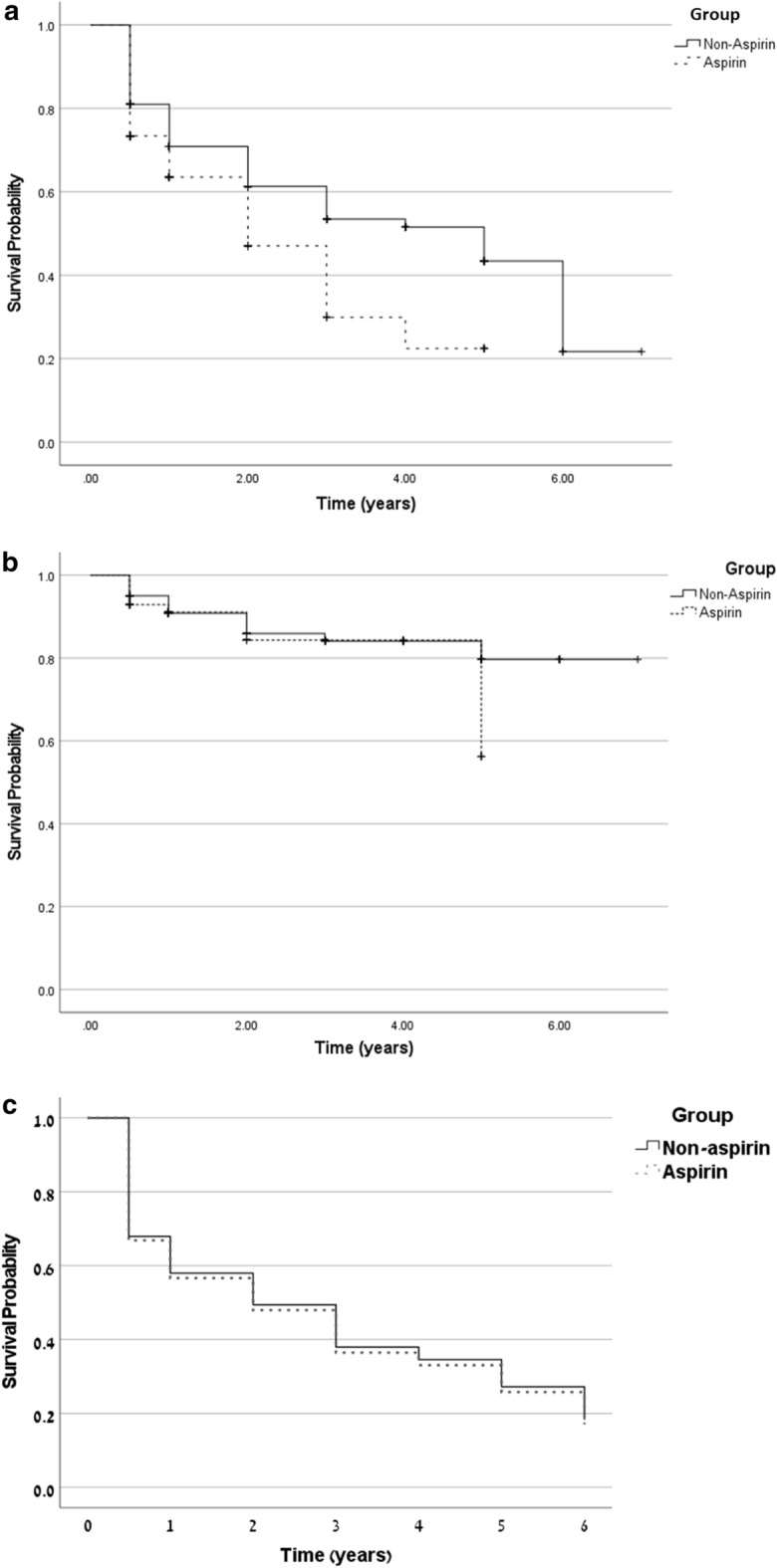
Cox proportional hazard regression models in aspirin versus non-aspirin users An adjusted Cox proportional hazard regression model was done after controlling diabetes, atrial fibrillation, age group and CKD group. (**a**) Cox regression survival curve for CVD. (**b**) Cox regression survival curve for bleeding. (**c**) Cox regression survival curve for all-cause mortality.

Cardiovascular events occurred in 566 patients (31.1%), most frequently Acute Coronary Syndrome (271 ;47.8% of cardiovascular events). On univariate analysis, cardiovascular events affected more subjects in the aspirin group than in the non-aspirin group (35% vs. 27.3%, respectively, RR = 1.43, *p* < 0.01; Table 2, Fig. 2). This difference was mainly driven by the higher occurrence of ACS among aspirin users. The RR for CVD events was higher among subjects with diabetes (Supplementary Table S2) . On multivariate analysis, aspiring use was not associated with CVD event rates, while diabetes remained significantly associated with increased risk for CVD (RR = 1.41 [1.10, 1.84], *p* < 0.05; Table S2). On Cox regression analysis of CVD events among aspirin users versus non users, there was no significant difference between the groups (Log rank *p* = 0.324, Fig. 3a).

**Table 2:** Differences in outcomes according to aspirin prescription.

Variable	Aspirin (N = 909)	Non-aspirin (N = 909)	Total (N = 1,818)	Chi-squared	Risk ratio [95% CI]	*p*-value
CVD	318 (35.0)	248 (27.3)	566 (31.1)	12.57	1.43 [1.17, 1.75]	< .01
**CVD type (% from total CVD events)**				2.91		.40
Stroke	119 (37.4)	104 (42)	223 (39.4)			
ACS	159 (50.0)	112 (45.2)	271 (47.9)			
PVD	40 (12.6)	32 (12.9)	72 (12.7)			
Bleeding	84 (9.2)	83 (9.1)	167 (9.2)	0.01	1.01 [0.73, 1.39]	.93
**Bleeding source**						
Gastrointestinal	54 (64.3)	58 (70.7)	112 (67.5)	0.15	0.92 [0.63, 1.35]	.77
Hemoptysis	6 (7.1)	3 (3.7)	9 (5.4)	1.01	2.00 [0.50, 8.04]	.51
Intra-cranial	11 (13.1)	9 (11.0)	20 (12.0)	0.20	1.22 [0.56, 2.70]	.65
Hematuria	12 (14.3)	11 (13.4)	23 (13.9)	0.04	1.09 [0.48, 2.48]	.83
Mixed	0	1 (1.2)	1 (0.6)	0.87	0.59 [0.53, 0.66]	.92
Epistaxis	1 (1.2)	0 (0)	1 (0.6)	–	–	–
All-cause mortality	371 (40.8)	395 (43.5)	766 (42.1)	1.29	0.89 [0.74, 1.08]	.25

Values are presented as absolute numbers (percentage).

*CVD* cardiovascular disease; *ACS* acute coronary syndrome; *PVD* peripheral vascular disease.

Rates of major bleeding were similar between groups (9.2% in aspirin users vs. 9.1% in non-users,* p* = *0.93,* Table 2). This was consistent in univariate and multivariate analyses (supplementary Table S2 and Fig. 3b ; Log rank *p* = 0.295). The most common bleeding source in both groups was gastrointestinal, with no significant difference in sources of major bleeding between aspirin users and non-users.

During the study period, 42.1% of the patients died. Mortality rates were comparable between aspirin users and non-users (Table 2). All-cause mortality was also similar between study groups after controlling for possible confounders using both regression models (supplementary Tables S2, S3). Age and advanced CKD stages were associated with increased overall mortality in multivariate logistic regression and Cox regression models (supplementary Tables S2, S3).

In an unadjusted log rank test, mean survival was numerically longer in the non-aspirin group versus the aspirin group. This difference was not statistically significant (3.2 vs. 2.2 years, *χ*^2^(1) = 2.14, *p* = 0.14). However, a Cox regression analysis showed no significant difference in all-cause mortality rates between aspirin users and nonusers after controlling for possible confounders, (OR = 1.01, CI[0.78, 1.92], *p* = 0.72).

Cox proportional hazard regression model was done after controlling diabetes, atrial fibrillation, age group and CKD group. The adjusted survival curves of this model are shown in Fig. 3c.

## Discussion

In the current study, based on real-life data, we did not find a survival benefit for chronic aspirin treatment among elderly patients with CKD without previous cardiovascular disease. Aspirin treatment is a well-established therapy for secondary prevention of cardiovascular disease, but its role in primary prevention is less-well validated in the general population and among specific sub-populations such as CKD patients, who are often excluded and not well-represented in clinical trials^1,7–9^.

This study included all patients with CKD who were either hospitalized or attended the ambulatory clinics at Meir Medical Center over 5 years, and continued follow-up for 2 additional years. The strict inclusion and exclusion criteria resulted in only 1,818 patients remaining from an initial cohort of 10,302.

Mean age was 73.4 years and 41.7% had severe CKD (eGFR < 30 ml/min/1.73m^2^). Most patients had comorbidities, such as hypertension and diabetes, as expected with elderly CKD patients. During the follow-up period, 42.1% of the patients died and 31.1% had at least one non-fatal cardiovascular event. These high rates of events are also expected in older patients with advanced CKD and comorbidities. While the overall mortality and cardiovascular event rates were higher than in previous studies, our results agree with the reported lack of benefit with aspirin for patients with severe CKD^14,15^.

High mortality rates among CKD patients were described in a cohort of almost 28,000 CKD patients, who were 10 times more likely to die than to progress to end stage renal disease. In this cohort, even a mildly decreased renal function was associated with significantly increased risk of mortality. Our study, which included patients with even lower baseline kidney function, supports these results^16^.

The main determinants of all-cause mortality in our study were age and advanced CKD, as was reported previously^17–22^.

Aspirin treatment did not result in a decrease in non-fatal cardiovascular events. This could be explained by the multifactorial nature of CVD among CKD patients, including unique risk-factors such as increased oxidative stress, endothelial dysfunction, elevated FGF23, and accelerated vascular calcifications. In addition, several studies reported a threefold increased frequency of aspirin resistance in CKD patients as compared to controls, in up to 46% of patients^17^. Patients resistant to aspirin are at greater risk for cardiovascular morbidity than those who are sensitive to aspirin^11^. This observation might partially explain the high prevalence of CVD and lack of effectiveness in primary prevention among CKD patients. Our finding are in contrast to the post-hoc analysis of the HOT study, which reported decreases in major cardiovascular events and mortality in hypertensive patients with CKD^18^, as well as other reports^16,17^.

The ASPREE trial reported no survival or CVD benefits among elderly patients treated with aspirin for primary prevention, compared with placebo. Median age in the trial was 74 years, similar to the age of participants in the current study. Thus, our results among CKD patients might reflect the futility of aspirin as primary prevention among subjects in this age group, in general^8,9^. In a recently published meta-analysis, aspirin treatment for primary prevention of CVD in patients with CKD did not provide any benefit for CVD and showed increased risk of bleeding^19^.

Rates of major bleeding in our study, most often gastrointestinal, were comparable between groups. We did not assess gastrointestinal protective medications, which may serve as potential confounders. Kim et al. found a higher rate of adverse events and harmful consequences in chronic aspirin users^3^, and an increased bleeding risk was reported in the ASPREE trial^10^. We found that hypertension and advanced kidney disease were associated with major bleeding among aspiring non-users only, as was also described in a meta-analysis on patients with diabetes and hypertension^20^.

CVD are leading causes of morbidity and mortality in the growing population of CKD patients, and research regarding lowering this risk is needed. Considering the opposite predisposition to thrombotic and bleeding events, the higher incidence of aspirin resistance and the currently limited available data, we believe that further investigation from the real-world concerning aspirin for primary prevention among CKD patients is warranted^21^. The expected ATTACK trial, scheduled for completion in 2025, could clarify this unresolved question^22^.

This study had several limitations inherent to the retrospective design. Aspirin users were older and suffered more from diabetes and atrial fibrillation. We used propensity score matching and multivariate models to control for confounders and selection bias. The modest sample size and high exclusion rates, might have contributed to bias, as well. We did not include subjects in whom CKD manifested without reduced eGFR (such as abnormal urinalysis, proteinuria, or anatomical defects). We also did not record albuminuria that is a well-established cardiovascular risk factor by itself^23^.

The heterogeneity of CKD population and multifactorial nature of cardiovascular disease among CKD patients may also contribute to potential confounders. This should prompt further research in this field. We believe aspirin use should be assessed on an individual basis depending on risk for CVD and bleeding.

## Supplementary Information

Supplementary Information.

## Data Availability

The data that support the findings of this study are available on request from the corresponding author, KCH.
