# Supplementary material for: Effect of aspirin on primary prevention of cardiovascular disease and mortality among patients with chronic kidney disease
Source: Sci Rep. 2022 Oct 22;12:17788. doi: 10.1038/s41598-022-22474-9 (PMC9588077; doi:10.1038/s41598-022-22474-9)
Supplement: Supplementary file 1 — Supplementary Information. [file 41598_2022_22474_MOESM1_ESM.docx]

**Supplementary Table 1:** Clinical and demographic characteristics of study population

|  | **Before propensity score matching** | | |  | **After propensity score matching** | | |
| --- | --- | --- | --- | --- | --- | --- | --- |
| **Characteristic** | **Aspirin**  **(N = 912)** | **Non-aspirin**  **(N = 1260)** | **P-value** |  | **Aspirin**  **(N = 909)** | **Non-aspirin**  **(N = 909)** | **P-value** |
| Sex |  |  | .65 |  |  |  | .57 |
| Male | 505 (55.6) | 688 (54.6) |  |  | 505 (55.6) | 493 (54.2) |  |
| Female | 407 (44.4) | 572 (45.4) |  |  | 404 (44.4) | 416 (45.8) |  |
| Age, years | 74.24±10.28 | 72.70±12.62 | .01 |  | 74.29±10.29 | 73.88±12.78 | .47 |
| Follow-up, years |  |  | .23 |  | 4.83±1.31 | 4.931±1.48 | .43 |
| First creatinine, mg/dL | 2.21±1.06 | 2.30±1.08 | .83 |  | 2.24±1.02 | 2.18±1.04 | .75 |
| Baseline eGFR, ml/min | 30.01±12.43 | 30.03±11.47 | .88 |  | 32.56±11.21 | 31.87±11.26 | .72 |
| CKD group |  |  | .79 |  |  |  | .76 |
| [Stage 3A](about:blank) | 79 (8.7) | 98 (7.8) |  |  | 79 (8.7) | 71 (7.8) |  |
| [Stage 3B](about:blank) | 366 (40.5) | 522 (41.5) |  |  | 366 (40.5) | 386 (42.5) |  |
| [Stage 4](about:blank) | 355 (39.3) | 485 (38.6) |  |  | 355 (39.3) | 342 (37.6) |  |
| [Stage 5](about:blank) | 103 (11.4) | 153 (12.2) |  |  | 103 (11.4) | 111 (12.2) |  |
| Hypertension | 790 (86.7) | 1079 (85.6) | .42 |  | 790 (86.9) | 778 (85.6) | .41 |
| Diabetes mellitus | 593 (65.1) | 662 (52.5) | <.01 |  | 593 (65.2) | 478 (52.6) | <.01 |
| Atrial fibrillation | 72 (7.8) | 46 (3.7) | <.01 |  | 71 (7.8) | 36 (4.0) | <.01 |

*Note:* Discrepancies between total number of patients, and number of patients for both groups are due to missing data

Values are presented as absolute numbers (percentage) or as mean ± SD

**Supplementary Table 2:** Multivariate logistic regression model showing odds ratios and confidence intervals for predicting mortality, bleeding and cardiovascular disease (CVD)

| **Variable** | **All-cause mortality** | **Bleeding** | **CVD** | |
| --- | --- | --- | --- | --- |
| Hypertension | 0.72 [0.52, 1.15] | 1.51 [0.83, 2.76] | 1.41 [0.98, 2.01] | |
| Diabetes mellitus | 1.11 [0.83, 1.42] | 0.81 [0.58, 1.17] | 1.41* [1.10, 1.84] | |
| Age | 3.12** [2.15, 5.15] | 1.15 [0.67, 1.95] | 1.13 [0.81. 1.65] | |
| CKD Group | CKD 3A - reference group | | |  |
| Stage 3B | 0.91 [0.40, 1.22] | 1.18 [0.62, 2.78] | 1.03 [0.82, 1.72] | |
| Stages 4 and 5 | 2.12** [1.21, 3.02] | 1.35 [0.66, 2.61] | 1.05 [0.67, 1.65] | |
| Aspirin | 1.03 [0.62, 1.84] | 1.09 [0.65, 1.72] | 1.05 [0.61, 3.14] | |

** p<0.05

**Supplementary Table 3: Cox regression model with hazard ratios and confidence intervals for predicting all-cause mortality, bleeding and CVD**

| **Variable** | **All-cause mortality** | **Bleeding** | **CVD** | |
| --- | --- | --- | --- | --- |
| Hypertension | 0.61 [0.48, 1.21] | 1.33 [0.78, 2.20] | 1.11 [0.86, 1.43] | |
| Diabetes mellitus | 1.10 [0.78, 1.34] | 0.86 [0.60, 1.13] | 1.14 [0.96, 1.37] | |
| Age | 3.10** [2.10, 5.02] | 1.01 [0.99, 1.02] | 1.01 [0.99. 1.01] | |
| CKD Group | CKD 3A - reference group | | |  |
| Stage 3B | 0.781 [0.35, 1.15] | 0.81 [0.48, 1.38] | 0.95 [0.71, 1.27] | |
| Stages 4 and 5 | 1.98** [1.10, 3.14] | 1.03 [0.60, 1.75] | 0.96 [0.71, 1.30] | |
| Aspirin | 1.01 [0.78, 1.92] | 0.75 [0.55, 1.03] | 0.98 [0.83, 1.17] | |

** p<0.05
